# Functions of the *Clostridium acetobutylicium *FabF and FabZ proteins in unsaturated fatty acid biosynthesis

**DOI:** 10.1186/1471-2180-9-119

**Published:** 2009-06-04

**Authors:** Lei Zhu, Juanli Cheng, Biao Luo, Saixiang Feng, Jinshui Lin, Shengbin Wang, John E Cronan, Haihong Wang

**Affiliations:** 1 College of Life Science, South China Agricultural University, Guangzhou 510642, PR China; 2 Department of Biochemistry, University of Illinois at Urbana-Champaign, Urbana, Illinois 61801, USA; 3 Department of Microbiology, University of Illinois at Urbana-Champaign, Urbana, Illinois 61801, USA

## Abstract

**Background:**

The original anaerobic unsaturated fatty acid biosynthesis pathway proposed by Goldfine and Bloch was based on in *vivo *labeling studies in *Clostridium butyricum *ATCC 6015 (now *C. beijerinckii*) but to date no dedicated unsaturated fatty acid biosynthetic enzyme has been identified in Clostridia. *C. acetobutylicium *synthesizes the same species of unsaturated fatty acids as *E. coli*, but lacks all of the known unsaturated fatty acid synthetic genes identified in *E. coli *and other bacteria. A possible explanation was that two enzymes of saturated fatty acid synthesis of *C. acetobutylicium*, FabZ and FabF might also function in the unsaturated arm of the pathway (a FabZ homologue is known to be an unsaturated fatty acid synthetic enzyme in enterococci).

**Results:**

We report that the FabF homologue located within the fatty acid biosynthetic gene cluster of *C. acetobutylicium *functions in synthesis of both unsaturated fatty acids and saturated fatty acids. Expression of this protein in *E. coli *functionally replaced both the FabB and FabF proteins of the host in *vivo *and replaced *E. coli *FabB in a defined in *vitro *fatty acid synthesis system. In contrast the single *C. acetobutylicium *FabZ homologue, although able to functionally replace *E. coli *FabZ in *vivo *and in *vitro*, was unable to replace FabA, the key dehydratase-isomerase of *E. coli *unsaturated fatty acid biosynthesis in *vivo *and lacked isomerase activity in *vitro*.

**Conclusion:**

Thus, *C. acetobutylicium *introduces the double of unsaturated fatty acids by use of a novel and unknown enzyme.

## Background

**Figure 1:**
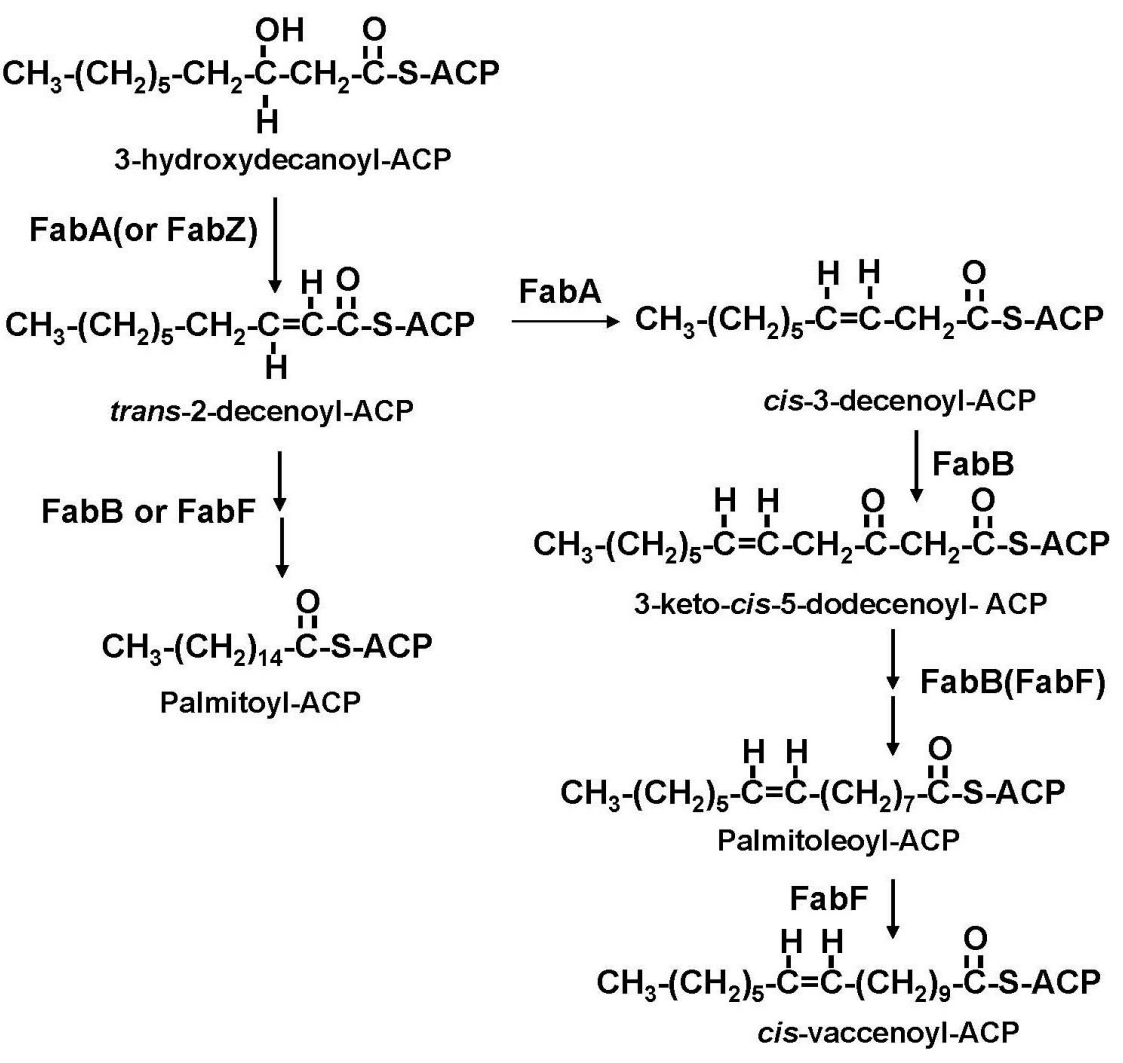
**Unsaturated fatty acid biosynthetic pathway of *E. coli***.

Bacterial growth requires an appreciable fraction of the acyl chains of the membrane lipids to be in a disordered state[1,2]. Such disordered states are imparted by fatty acids that act to offset the closely packed ordered arrangement of the lipid bilayer acyl chains imparted by straight-chain saturated acyl chains. In most bacteria the role of introducing acyl chain disorder is fulfilled by unsaturated fatty acids (UFAs). Some bacteria synthesize UFA by desaturation, an oxygen-requiring reaction that introduces the double bond in a single concerted reaction [2]. However, as first recognized by Bloch and coworkers this is not an option for anaerobically grown bacteria [3]. These investigators originally proposed that introduction of the double bond involved a direct dehydration of the 3-hydroxydecanoyl intermediate of fatty acid synthesis to give a *cis*-3 double bond which would be conserved though subsequent cycles of addition of two carbon atoms to give the membrane lipid UFA moieties [4]. However, when tested in cell-free extracts of *E. coli*, the reaction proved to proceed by a more conservative dehydration to give the classical *trans*-2-decenoyl fatty acid synthetic intermediate followed by isomerization of the *trans*-2-double bond to the *cis*-3 species [3,5]. This *cis *double bond was then preserved through successive C_2 _elongation cycles to form the double bond of the mature UFAs [6,7]. The dehydration and isomerization reactions were demonstrated by purification of the *E. coli *FabA enzyme (called the "Bloch dehydratase" to distinguish it from the *E. coli *FabZ dehydratase of the elongation cycle) that catalyzed both the dehydration and isomerization reactions(Fig. 1) [5]. Ironically, although the pathway was originally proposed based on the patterns of incorporation of short chain radioactive fatty acids into UFAs by cultures of *Clostridium butyricum *(now *Clostridium beijerinckii*) [4], all of the extant Clostridial genomes lack a homologue of FabA, the *E. coli *dehydratase-isomerase studied by Bloch and coworkers. Indeed, many bacterial genomes do not encode a recognizable FabA. This is also true of FabB, the *E. coli *chain elongation enzyme that channels the metabolic intermediate produced by FabA into the mainstream fatty acid synthetic pathway. Indeed in the extant genome sequences FabA and FabB homologues are encoded only in the genomes of α- and γ-proteobacteria [6,7]. Thus far, two solutions that solve the problem of anaerobic UFA synthesis in the absence of FabA and FabB have been reported. The first solution was that of *Streptococcus pneumoniae *which introduces a *cis *double bond into the growing acyl chain using FabM, a *trans*-2 to *cis*-3-decenoyl-ACP isomerase (i.e., the second partial reaction of FabA) [8]. The second solution was that of *Enterococcus faecalis *which uses homologues of FabZ and FabF to perform the functions performed by FabA and FabB in *E. coli *[9]. *E. faecalis *encodes two FabZ homologues and two FabF homologues (FabF is closely related to FabB). Wang and Cronan [9] showed that one of these proteins, now called FabN, functioned as a dehydratase/isomerase analogous to FabA, whereas the other FabZ homologue possessed only dehydratase activity. A similar picture was seen for the FabF proteins, one (now called FabO) performed the FabB function whereas the other functioned only as a FabF [9]. However, neither of these scenarios seemed applicable to the Clostridia. *C. acetobutylicium *lacks *fabM, fabA *and *fabB *and has only a single copy of *fabZ*, although its fatty acid composition is similar to that of *E. coli*. This bacterium contains three genes that encode putative FabFs, although only one of these seemed likely to be involved in fatty acid synthesis (see Discussion). The most likely FabF homologue candidate was that encoded within a large gene cluster (*fabH acpP fabK*, *fabD fabG fabF accB fabZ accC accD accA*) that encodes what appears to be a complete set of the genes required for saturated fatty acid synthesis. How does *C. acetobutylicium *make unsaturated fatty acids? One possibility was that the single FabZ and FabF homologues could somehow function in both the saturated and unsaturated branches of the fatty acid synthetic pathway. We report that the *C. acetobutylicium *FabZ cannot catalyze isomerization of its *trans*-2-decenoyl-ACP product to the *cis-*3 species either in *vitro *or when expressed in *E. coli*. However, the single FabF homologue active in fatty acid synthesis has the functions of both *E. coli *long chain 3-ketoacyl-ACP synthases, FabB and FabF.

## Results

### Only one of the three *C. acetobutylicium fabF *homologues can functionally replace *E. coli *FabF in *vivo*

**Figure 2:**
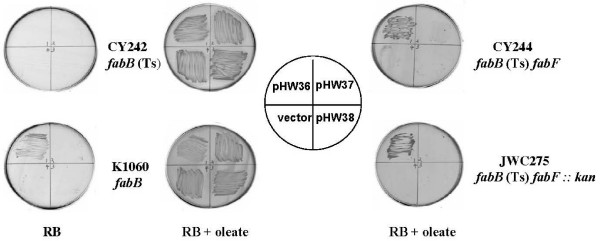
**Growth of *E. coli *strains CY242, K1060, CY244, and JWC275 transformed with plasmids encoding the *C. acetobutylicium fabF *homologues**. Following induction by addition of arabinose, transformants of strain K1060 were grown at 37°C, whereas the transformants of strains CY242, strain CY244 and strain JWC275 were grown at 42°C. The strains carried plasmids pHW36, pHW37 or pHW38 encoding *fabF1, fabF2 *and *fabF3*, respectively, or the vector plasmid, pBAD24.

There are three annotated *C. acetobutylicium fabF *homologues designated as CAC3573, CAC2008 and CAA0093 [10]. We will temporarily call these genes *fabF1, fabF2 *and *fabF3*, although our data indicate that only the first of these genes functions in fatty acid synthesis. To test the functions of these homologues, the three genes were inserted into the arabinose-inducible vector pBAD24. The resulting plasmids were then introduced into two *E. coli fabB*(Ts) *fabF *strains, CY244 and JWC275. At the non-permissive temperature these mutant strains lack both long chain 3-ketoacyl-ACP synthase activities and thus are unable to grow even when the medium is supplemented with the unsaturated fatty acid, oleate [11,12]. Derivatives of strains CY244 or JWC275 carrying pHW36 encoding *fabF1 *grew at 42°C in the presence of oleate whereas the strains carrying pHW37 and pHW38 (encoding *fabF2 *and *fabF3*, respectively) failed to grow (Fig. 2) (similar results were seen with plasmids of both low and high copy number vectors). Thus, only *fabF1 *complemented the *E. coli fabF *mutation showing that *C. acetobutylicium *FabF1, like *E. coli *FabF, is able to catalyze all of the elongation reactions required in the synthesis of saturated fatty acids. Furthermore, expression of FabF1 restored thermal control of fatty acid composition to a FabF null mutant strain (Table 1). An *E. coli fabF *strain in which *C. acetobutylicium *FabF1 was expressed from the *lac *promoter of a low copy number vector closely mimicked the changes in fatty acid composition seen in wild type *E. coli *strains upon changes in growth temperature [13]. Expression of FabF1 restored *cis*-vaccenate synthesis at all temperatures, but was much more effective at 30°C than at 37°C or 42°C (Table 1). This effect seems likely to be due to the effects of temperature on FabF1 synthase activity since thermal regulation disappeared upon expression of FabF1 from a high copy number vector (Table 1) and the enzyme was thermolabile in *vitro *(see below). Apparently, at high growth temperatures low levels FabF1 elongation activity was overcome by high-level expression of the protein. We also found high levels of *cis*-vaccenate at the non-permissive temperature upon expression of *fabF1 *in an *E. coli fabB fabF *strain that carried the *fabB *gene of *Haemophilus influenzae*, a bacterium naturally defective in both *cis*-vaccenate synthesis and in regulation of fatty acid composition by temperature [14] (data not shown).

**Table 1:** Effects of growth temperature on fatty acid compositions (% by weight)of *fabF *strain MR52 carrying plasmids encoding *C. acetobutylicium fabF1*.

	30°C		37°C		42°C	
	
Fatty acid	pHW33	pHW36	pHW33	pHW36	pHW33	pHW36
C14:0	2.2	5.8	2.4	6.2	2.6	3.3
C16:1	40.3	29	35	24.8	53.4	28.9
C16:0	21.4	25.8	32.4	25.1	26.2	28.7
C18:1	33.3	30	25.9	32.4	14.8	30.2
C18:0	2.8	9.4	4.3	11.6	2.9	8.7

### The *C. acetobutylicium fabF1 *gene can functionally replace *E. coli *FabB

**Figure 3:**
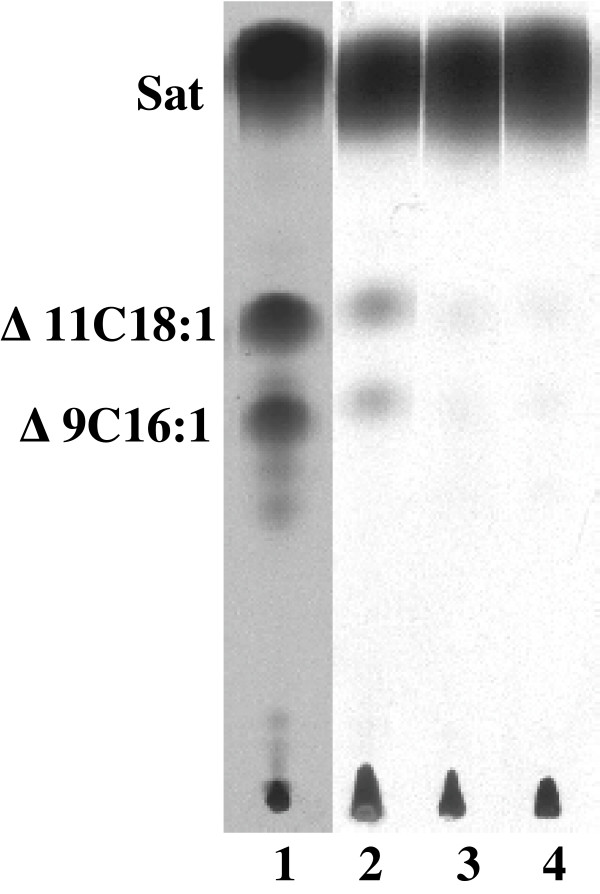
**Expression of *C. acetobutylicium *FabF1 restores UFA synthesis to *E. coli fabB *strains**. The methyl esters of fatty acids were obtained from the phospholipids as described in Methods. Lane 1 is the esters of the wild type *E. coli strain *MG1655. Lane 2 is the esters of strain CY242 carrying pHW36 (*fabF1*) in presence of arabinose induction. Lane 3 is the esters of strain CY242 carrying pHW36 (*fabF1*) in the absence of induction. Lane 4 is the esters of strain CY242 carrying vector pBAD24. The migration positions of the methyl esters of the fatty acid species are shown. The designations are: Sat, saturated fatty acid esterss; Δ9C16:1, methyl ester of *cis*-9-hexadecenoic; Δ11C18:1, methyl ester of *cis*-11-octadecenoic.

Although the presence of plasmid pHW36 (*fabF1*) allowed growth of the two *E. coli fabB*(Ts) *fabF *strains at the non-permissive temperature, growth of both strains required oleate. The lack of growth in the absence of oleate argued that either FabF1 lacked the ability to replace FabB or that FabF1 was unable to simultaneously perform the tasks of both FabB and FabF under these conditions. To decide between these alternatives we transformed pHW36 into strain K1060, a strain that carries an unconditional *fabB *allele, and into strain CY242 which carries the same *fabB*(Ts) allele as strain CY244. The complementation experiments showed that *C. acetobutylicium fabF*1 allowed strain K1060 to grow on RB medium lacking oleate at 37°C (Fig. 2). However, *fabF*1 failed to complement growth of the temperature sensitive *fabB *mutant strain, CY242 at 42°C (Fig. 2). If FabF1 possessed FabB activity at 37°C, unsaturated fatty acids should be synthesized. To test this hypothesis, we grew strain K1060 carrying pHW36 or pHW33 (both encoding *fabF1*) at different temperatures and the fatty acid compositions of these strains were determined by collision induced dissociation electrospray mass spectroscopy (CID ES-MS) (Table 2). The strains clearly synthesized unsaturated fatty acids when grown at all of the different temperatures. However, the level of unsaturated fatty acids synthesized was lower than that seen in K1060 carrying a plasmid (pCY9) that encoded *E. coli fabB *and the amount of *cis*-vaccenate decreased with increased growth temperature. Moreover, despite the differing copy numbers, the two plasmids that encoded *C. acetobutylicium *FabF1 gave similar levels of unsaturated fatty acids. These results provide an explanation for lack of complementation of the *fabB*(Ts) phenotype at 42°C by the *fabF1*-encoding plasmids. At 42°C the low activity of FabF1 did not allow enough unsaturated fatty acid synthesis to support growth. To test whether or not *C. acetobutylicium *FabF1 has FabB function at 42°C we assayed unsaturated fatty acid synthesis in strain CY242 carrying the *fabF*1 plasmid pHW36 (growth was supported by cyclopropane fatty acid supplementation) (Fig. 3). Under these conditions [^14^C] acetate labeling showed low levels of unsaturated fatty acids synthesis upon arabinose induction of FabF1 expression (Fig. 3). Therefore, FabF1 has the ability to replace FabB in *E. coli *unsaturated fatty acid synthesis but its expression allows growth only when the host FabF is present to perform the bulk of the chain elongation reactions.

**Table 2:** Fatty acid compositions (% by weight)of *fabB *strain K1060 transformed with plasmids encoding either *C. acetobutylicium fabF1 *or *E. coli fabB*.

	30°C			37°C			42°C		
	
Fatty acid	pHW33	pHW36	pCY9	pHW33	pHW36	pCY9	pHW33	pHW36	pCY9
C14:0	4.9	9.2	2.2	11.1	7.7	4	11.1	9.9	2.5
C16:1	12.8	8.1	16.8	17.5	18	20	19.7	13.5	20.3
C16:0	22.1	21.6	10.8	25.9	23.6	13.8	32.6	42.7	19.7
C18:1	43.1	43.1	67.1	31.8	34.4	58.1	17.7	22.4	51
C18:0	17	18	3.2	13.7	16.3	3.7	18.9	11.5	6.5

### Functional analysis of *C. acetobutylicium *FabZ in *vivo*

**Figure 4:**
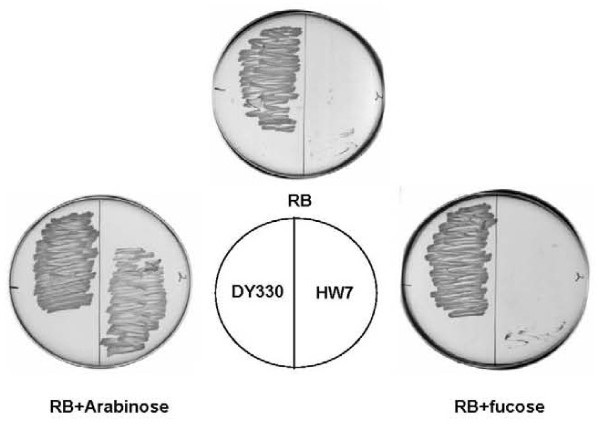
**Growth of *E. coli fabZ *mutant strain HW7 carrying plasmid pHW22 encoding *C. acetobutylicium fabZ***. The plates were of RB medium ei ther unsupplemented or supplemented with the inducer, L-arabinose, or supplemented with the anti-inducer, D-fucose, as shown. The plates were incubated at 30°C. Strain DY330 has the wild type *fabZ *locus whereas strain HW7 is Δ*fabZ*.

**Figure 5:**
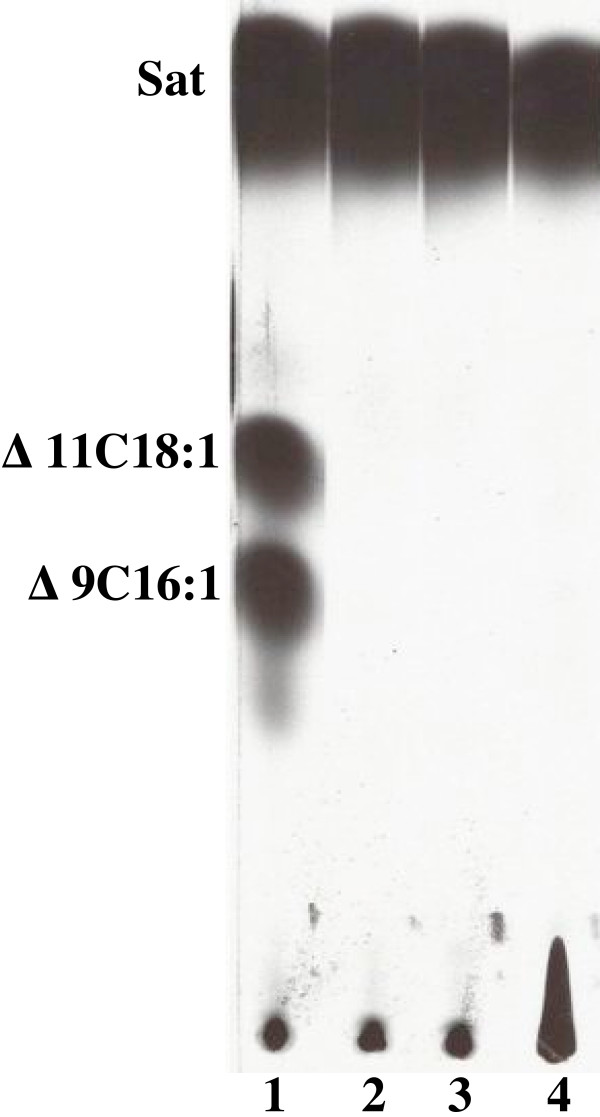
**Argentation thin-layer chromatographic analysis of [1-^14^C] acetate-labeled esters from strain CY57 transformed with pHW22 encoding *C. acetobutylicium fabZ***. The methyl esters of fatty acids were obtained from the phospholipids as described in Methods. Lane 1 is the methyl esters of the wild type *E. coli *strainMG1655. Lane 2 is the esters of strain CY57 carrying vector pBAD24. Lane 3 is the esters of strain CY57 carrying pHW22 which encodes the *C. acetobutylicium fabZ *labeled in the absence of induction. Lane 4 is the esters of strain CY57 (pHW22) following arabinose induction. Labels are as in Fig. 2.

The sole *fabZ *homologue in the *C. acetobutylicium *genome is located within a large cluster of putative *fab *genes [10]. To test function of the encoded protein the *fabZ *gene was inserted into the arabinose-inducible pBAD24 expression vector to give plasmid pHW22. Since *E. coli fabZ *null strains are nonviable [15,16], we first introduced pHW22 into strain DY330, a "recombineering" strain [17]. We then expressed the *C. acetobutylicium *FabZ in this strain and used standard phage γ recombinase manipulations to delete the host *fabZ *gene. These manipulations gave strain HW7, which grew well in presence of arabinose but failed to grow in the presence of fucose, an anti-inducer of arabinose promoter expression (Fig. 4). The fatty acid composition of the complemented mutant strain grown in presence of arabinose was similar to that of the parental strain, DY330, indicating that *C. acetobutylicium *FabZ functionally replaced *E. coli *FabZ (Table 3). The lack of *fabA *and *fabM *homologues in *C. acetobutylicium *raised the possibility that the FabZ of this organism might function as both an isomerase and a dehydratase as does the *E. faecalis *FabZ-like protein, FabN [9]. To test this possibility plasmid pHW22 was introduced into both the *fabA*(Ts) *E. coli *strain CY57 and the *fabA *null mutant strain MH121. Neither stain grew in the absence of unsaturated fatty acid supplementation (data not shown) indicating that *C. acetobutylicium *FabZ lacks isomerase function and thus was unable to functionally replace FabA. However, it remained possible that *C. acetobutylicium *FabZ catalyzed UFA synthesis, but that the levels of UFA produced were too low to support growth. This possibility was tested by [^14^C]-acetate labeling of the fatty acids synthesized by strain CY57 carrying pHW22 and analysis of the resulting radioactive fatty acids for traces of UFA (Fig. 5). No UFA synthesis was detected. Another possible explanation for the observed lack of UFA synthesis was that FabI, the enoyl-ACP reductase of *E. coli*, converted the intermediate *trans*-2-decenoyl-ACP to decanoyl-ACP before the putative isomerase activity of *C. acetobutylicium *FabZ could act. Thus, we repeated the labeling experiment in the presence of a low dose of triclosan, a specific *E. coli *FabI inhibitor [6], in order to give the putative isomerase a better opportunity to act on the *trans*-2-decenoyl-ACP intermediate. Again no synthesis of unsaturated fatty acid was observed (data not shown). These *in vivo *results argued strongly that that *C. acetobutylicium *FabZ was unable to isomerize *trans*-2-decenoyl-ACP.

**Table 3:** Composition of fatty acids of strain HW7

	Fatty acid composition (% by weight)			
	
	C14:0	C16:1	C16:0	C18:1
DY330	3.2	41.0	29.7	26.0
HW7	<0.5	49.6	29.2	21.2

### In *vitro *assay of *C. acetobutylicium *FabZ and FabF1 activities

**Figure 6:**
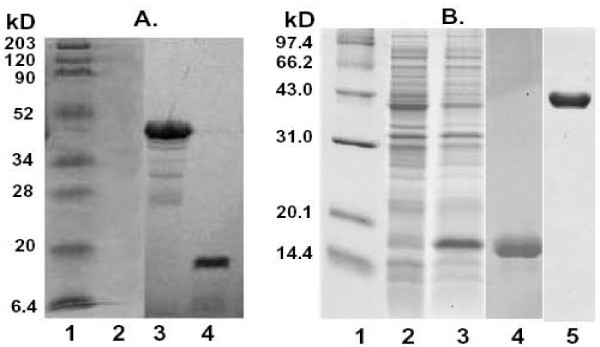
**Expression of *C. acetobutylicium *FabZ and FabF1 in *E. coli***. Panel A. Expression of *C. acetobutylicium *FabF1 and FabZ from their native coding sequences was induced in *E. coli *BL21(DE3) under control of a phage T7 promoter. Lane: 1, molecular mass markers; lane 2, proteins expressed in the presence of vector pET28b; lane 3, proteins expressed in the presence of pHW28 (FabF1) and lane 4, proteins expressed in the presence of pHW39 (FabZ). Panel B. An expression plasmid encoding the codon-optimized *C. acetobutylicium fabZ *was introduced into *E. coli *BL21 (DE3). Lane: 1, molecular mass markers; lane 2, plasmid pHW74 which expresses native *fabZ*; lane 3, plasmid pHW74m which expresses the codon-optimized *fabZ*; lane 4, FabZ expressed from the codon-optimized gene purified by nickel-chelate chromatography and lane 5, FabF1 purified by nickel-chelate chromatography.

**Figure 7:**
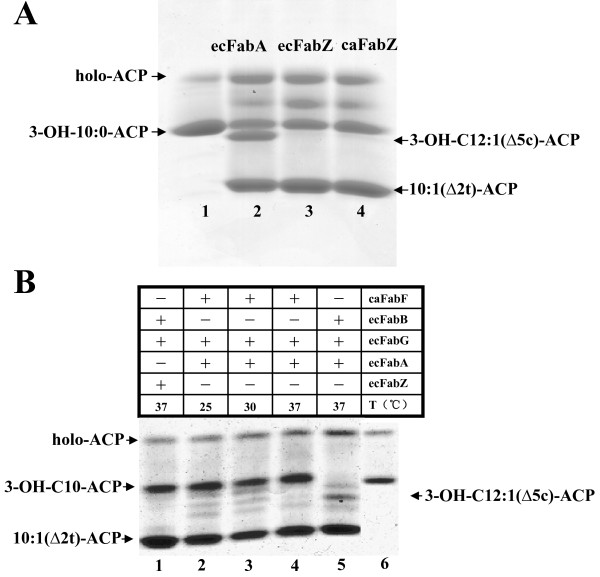
**Properties of *C. acetobutylicium *FabZ and FabF1 *in vitro***. Panel A. The ability of *C. acetobutylicium *FabZ to synthesize fatty acids was determined by conformationally-sensitive gel electrophoresis. Lanes: lane 1, no addition; lane 2, *E. coli *FabA (ecFabA) was added; lane 3, *E. coli *FabZ (ecFabZ) was added and lane 4, *C. acetobutylicium *FabZ (caFabZ) was added. Panel B. The reactions shown above the gel were as in lane 2 except that *E. coli *FabB was replaced with *C. acetobutylicium *FabF1 (caFabF) in lanes 2–4. Lane 6 is the 3-hydroxydecanoyl-ACP standard as in lane 1 of panel A.

To allow direct assay of *C. acetobutylicium *FabF1 and FabZ activities we expressed the proteins in *E. coli *to facilitate their purification. [^35^S]Methionine labeling showed that strain BL21 (DE3) carrying plasmids encoding either *C. acetobutylicium fabF1 *or *fabZ *under control of a phage T7 promoter expressed proteins of the expected sizes (Fig. 6A). However, the expression level of the FabZ protein was so low that it was not detected upon staining the SDS gels (Fig. 6B). We attributed this poor expression to the fact that the *C. acetobutylicium FabZ *gene contains 24 codons that correspond to nonabundant (rare) tRNA species in *E. coli*. We therefore changed these codons to synonymous codons that correspond to abundant *E. coli *tRNA species thereby resulting in a modified gene we call *fabZ*m. Plasmid pHW74m (which encoded the His-tagged *fabZ*m under T7 promoter control) abundantly expressed a protein with an apparent mass of 17 kDa (Fig. 6B) in good agreement with the expected value for the His_6_-tagged protein (17.5 kDa). The His_6_-tagged FabZ protein was purified to essential homogeneity using nickel-chelate chromatography (Fig. 6B). We also purified the N-terminally His_6_-tagged versions of *C. acetobutylicium *FabF1 and the *E. coli *fatty acid biosynthetic proteins FabD, FabG, FabA, FabZ, FabB and FabI plus the *Vibrio harveyi *AasS acyl-ACP synthetase [18] by nickel-chelate chromatography. AasS was used to synthesize the 3-hydroxydecanoyl-ACP substrate whereas the other enzymes were used to assemble a defined in *vitro *fatty acid synthesis system in which the activities of *E. coli *FabA and *C. acetobutylicium *FabZ or *E. coli *FabB and *C. acetobutylicium *FabF1 could be directly compared. In reactions containing FabA 3-hydroxydecanoyl-ACP was converted to a mixture of *trans*-2 and *cis*-3-decenoyl-ACPs as expected from prior work [19,20]. *E. coli *FabB is unable to elongate *trans*-2-decenoyl-ACP, but elongates the *cis*-3 species to 3-keto-*cis*-5-dodecenoyl-ACP in the presence of malonyl-ACP [20]. This product is then reduced by FabG and dehydrated by FabA to form *trans*-2-*cis*-5-dodecadienoyl-ACP[20]. The *trans*-2-*cis*-5-dodecadienoyl-ACP product accumulates because the reaction mixtures lacked enoyl-ACP reductase which precluded further elongations [20]. Using this system we showed (as has long been known) that FabA is a bifunctional enzyme that catalyzes both the dehydration of 3-hydroxydecanoyl-ACP and well as the reversible isomerization of *trans*-2-decenoyl-ACP to *cis*-3- decenoyl-ACP (Fig. 7). As expected *E. coli *FabZ converted 3-hydroxydecanoyl-ACP to *trans*-2-decenoyl-ACP. However, addition of *E. coli *FabB to this reaction failed to give the 12-carbon unsaturated elongation product seen with FabA (Fig. 7) in agreement with prior reports that *E. coli *FabZ acts solely as a dehydratase and that FabB is unable to elongate *trans*-2-decenoyl-ACP [20]. If *C. acetobutylicium *FabZ was capable of the isomerization reaction, then upon addition of *E. coli *FabB the reaction would yield *trans*-2, *cis*-5-dodecadienoyl-ACP [20]. However, the only product formed was *trans*-2-decenoyl-ACP, the product of *E. coli *FabZ (Fig. 7A). Hence, we conclude that *C. acetobutylicium *FabZ possesses only dehydratase activity and introduction of the *cis *double bond requires another enzyme that has yet to be discovered. In parallel experiments, we replaced *E. coli *FabB with *C. acetobutylicium *FabF1 in the *E. coli *FabA reaction mixture to test if *C. acetobutylicium *FabF1 could elongate *cis*-3-decenoyl-ACP (Fig. 7B). We found that addition of FabF1 gave a modest conversion of *cis*-3-decenoyl-ACP to *trans*-2-*cis*-5-dodecadienoyl-ACP and at 37°C the product yields were lower than those seen at 25°C and 30°C consistent with the low activity of FabF1 at high temperature seen in *vivo *(Fig 7B).

## Discussion

Although *C. acetobutylicium*, *C. beijerinckii *and *E. coli *synthesize the same species of unsaturated fatty acids [21] and Clostridia are thought to follow the same synthetic mechanism as *E. coli *[22], the enzyme that introduces the *cis *double bond of the unsaturated fatty acids remains unknown. Like other Clostridia the *C*.*acetobutylicium *genome encodes none of the three known anaerobic unsaturated fatty acid synthesis pathways denoted by the presence of genes encoding FabM, FabA or FabN proteins. One possibility was that the single FabZ of this bacterium could somehow partition acyl chains between the saturated and unsaturated branches of the pathway. However, our in *vivo *and in *vitro *data show that *C. acetobutylicium *FabZ cannot synthesize the first intermediate in unsaturated fatty acid synthesis. Hence, Clostridia must contain a novel enzyme that introduces the *cis *double bond. Note that the proposed isomerase activity of the *C. acetobutylicium *FabZ was not unreasonable. *C. acetobutylicium *FabZ shares 51.4 and 59.3% identical residues with *E. faecalis *FabN and FabZ, respectively, and there is no sequence signature that denotes isomerase ability [9,23,24]. This is because the isomerase potential of 3-hydroxyacyl-ACP dehydratases is not determined by the catalytic machinery at the active site but rather by the β-sheets that dictate the orientation of the central α-helix and thus the shape of the substrate binding tunnel [23,24]. We are currently seeking the gene(s) that encode the enzyme responsible for *cis *double bond introduction in *C. acetobutylicium*.

In contrast to FabZ, the single 3-ketoacyl-ACP synthase (FabF) of this bacterium performs the elongation functions required in both branches of the fatty acid synthetic pathway. This protein can both elongate palmitoleoyl-ACP to *cis-*vaccenoyl-ACP as does FabF in *E. coli *and also elongates the *cis *double bond containing product of FabA as does *E. coli *FabB. However, *C. acetobutylicium *FabF, was unable to perform the two tasks simultaneously and thus differs from *Enterococcus faecalis *FabO [9]. Although the *C. acetobutylicium *FabF and *E. faecalis *FabO proteins are 45–46% identical to *E. coli *FabF, they are only 55% identical to one another. Hence, each of the three proteins is distinct from the other two. The finding that *C. acetobutylicium *FabF was unable to perform the two tasks simultaneously could be due to the intrinsic temperature sensitivity of FabF1 and to the enzyme undergoing a type of kinetic confusion in this unnatural setting. Perhaps the intermediates of one branch of the pathway act (in effect) as inhibitors of the other branch. In this scenario the presence of the *E. coli *enzyme (either FabB or FabF) would result in the inhibitory intermediates being converted to long chain acyl chains, thereby freeing the *C. acetobutylicium *FabF to operate in the other branch. The complex task faced by FabF1 upon expression in an *E. coli *strain lacking both FabB and FabF is illustrated by the effects of overproduction of FabA and FabB in *E. coli *[25]. Overproduction of FabA results in increased production of saturated fatty acids rather than the increase in unsaturated fatty acid levels that might have been expected [25]. In contrast overproduction of FabB has the opposite result; unsaturated fatty acid levels are increased [25]. However, if the two enzymes are simultaneously overproduced, the fatty acid composition returns to normal [25]. These counter-intuitive results are due to the fact that FabA catalyzes reversible reactions whereas the FabB reaction is irreversible. Hence, when FabB activity is limiting, any excess *cis*-3-decenoyl-ACP produced by FabA can be isomerized back to *trans*-2-decenoyl-ACP and upon FabI action, this acyl chain can enter the saturated arm of the pathway. However, when FabB is in excess, it catalyzes the irreversible elongation of *cis*-3-decenoyl-ACP and thereby pulls the flow of carbon toward the unsaturated branch of the pathway. Thus, it would seem a surprising finding if the *C. acetobutylicium *FabF was able to accurately partition acyl chains between the two branches of the fatty acid synthetic pathway of a foreign organism.

It should be noted that it was not unexpected that the FabF homologue encoded within the *fab *gene cluster was the only FabF homologue that functioned in fatty acid synthesis. There are good arguments against the other two homologues having this function. The CAC2008 ORF in located within a cluster of genes that appear involved in synthesis of a glycosylated product of a hybrid polyketide-nonribosomal polypeptide pathway. If so, the CAC2008 ORF would be involved in synthesis of the polyketide moiety. The CAA0088 ORF is encoded on the *C. acetobutylicium *megaplasmid required for the late steps of solvent production by this organism. *C. acetobutylicium *survives loss of the megaplasmid [26] and therefore the CAA0088 ORF cannot encode an enzyme essential for fatty acid synthesis (although it could still provide FabF function). Note that it has been recently reported that the single FabF protein of the distantly related gram positive bacterium *Lactococcus lactis *can also perform the FabB reaction as well as that of FabF[27].

## Conclusion

Unsaturated fatty acid synthesis in Clostridia cannot be explained by a plenipotent FabZ indicating that these bacteria encode a novel enzyme that introduces the *cis *double bond. In contrast the Clostridia FabF protein has the functions of both of the long chain 3-ketroacyl-ACP syntheases of *E. coli*. The diversity of bacterial enzymes used for synthesis of the *cis *double bond of unsaturated fatty acids is unexpected because the remainder of the fatty acid synthetic enzymes is well conserved among very diverse bacteria.

## Methods

### Bacterial strains, plasmids and growth conditions

The *E. coli *strains and plasmids used in this study are listed in Additional file 1. Luria-Bertani medium was used as the rich medium for *E. coli*. The phenotypes of *fab *strains were assessed on rich broth (RB) medium [12]. Oleate neutralized with KOH was added to RB medium at final concentration of 0.1% and solubilized by addition of Brij 58 detergent to final concentration of 0.1 to 0.2%. Antibiotics were used at the following concentrations (in mg/L) sodium ampicillin, 100; chloramphenicol, 30; kanamycin sulfate and rifampicin, 200. L-Arabinose and D-fucose were used at concentrations of 0.01%. Isopropyl-β-D-thiogalactoside (IPTG) was used at final concentration of 1 mM.

### Recombinant DNA techniques and construction of plasmids

Restriction enzymes, T4 DNA ligase and Taq DNA polymerase were from Invitrogen or New England Biolabs unless indicated otherwise. All enzymatic reactions were carried out according to the manufacturer's specifications. Qiagen products were used to isolate plasmids, purify DNA fragments from agarose gels and purify PCR products. Plasmids were introduced into *E. coli *strains by CaCl_2_-mediated transformation. C. acetobutylicium ATCC824 genomic DNA was extracted using the GNOME DNA kit (Bio 101). DNA sequencing and the synthesis of oligonucleotides were done at the University of Illinois Keck Genomics Center.

The *C. acetobutylicium fabF *homologues were amplified from genomic DNA using the primers *fabF1, fabF2 *and *fabF3 *(Additional file 1). The PCR products were cloned into vector pCR2.1TOPO to give plasmids pHW40 (*fabF1*), pHW41 (*fabF2*) and pHW42 (*fabF3*). Plasmids pHW40 and pHW42 were then digested with EcoRI, the appropriate fragments were isolated and these were ligated into pHSG576 [28] digested with the same enzyme to give plasmids pHW33 and pHW35, respectively. The orientation of the *C. acetobutylicium *ORFs in these plasmids were such that the genes would be transcribed by the vector *lac *promoter. The HindIII-XhoI fragment of pHW41 was ligated into vector pSU20 [29] digested with the same enzymes to give pHW43 which was then digested with HindIII plus SalI and the *fabF2*-containing fragment was inserted into the same sites of vector pHSG576 to give pHW34. Plasmids pHW16, pHW31 and pHW32 were constructed as follows. The upstream primers were primers12, 34 and 56 (Additional file 1) and the downstream primer was the M13 (-) forward primer. Plasmids pHW33, pHW34 and pHW35 were used as templates for PCR amplification. The products were cloned into vector pCR2.1 TOPO to yield pHW16, pHW31 and pHW32, respectively. The BspHI-PstI fragments of pHW16 and pHW32 were then ligated into NcoI and PstI sites of pBAD24 [30] to give plasmids pHW36 and pHW38, respectively. Likewise, the BspHI-HindIII fragment of pHW31 was inserted into the NcoI and HindIII sites of pBAD24 to yield pHW37.

The *fabZ *homologue was amplified by PCR using *C. acetobutylicium *genomic DNA as template with primers Zprimer1 and Zprimer2 (Additional file 1). The PCR product was inserted into pCR2.1 TOPO vector to give pHW15. The BspLU11I-HindIII fragment of pHW15 was inserted into the sites of pBAD24 digested with NcoI and HindIII to give pHW22. The BspHI-EcoRI fragments of pHW15 and pHW16 was inserted into the NcoI and EcoRI sites of pET28b to give pHW39 and pHW28, respectively. By site-directed mutagenesis using the primers listed in Additional file 1, an NdeI site was introduced into pHW39 and pHW28. The NdeI-EcoRI fragment of this two new plasmids were inserted into the NdeI and EcoRI sites of pET28b to give pHW74 and pHW76. To increase FabZ expression, 24 codons that correspond to rare *E. coli *tRNA species were substituted with codons favored in *E. coli *by site-directed mutagenesis using the primers listed in Additional file 1 to give pHW74m. The NcoI-HindIII fragment of pHW74m was inserted into the NcoI and HindIII sites of pBAD24 to give pHW22m.

### Construction of an *E. coli fabZ *Deletion Strain

A linear DNA fragments carrying a *kan *cassette was amplified from pKD13 by PCR [9,31] using primers, HZ1 and HZ2 listed in Additional file 1. These primers were homologous at the 3' end for priming sequences in pKD13 and contained 45-nucleotide extensions at the 5' end homologous to the *E. coli fabZ *sequence. The 1.4 kb PCR product was purified, treated with DpnI, and then introduced into a pHW22-containing derivative of DY330 a strain lysogenic for a defective prophage that contains the recombination genes under control of temperature-sensitive *c*I-repressor [9]. The transformed cells were spread on LB plates containing ampicillin, kanamycin and arabinose. The *E. coli fabZ *deletion strain, HW7, was verified by PCR using primers P1, P2 plus HZ1, and HZ2.

### Analysis of phospholipid fatty acid compositions

Cultures (5 ml) were grown aerobically at different temperatures in RB medium overnight. The cells were then harvested and the phospholipids extracted as described previously [14]. The fatty acid compositions were analyzed by mass spectroscopy as described previously [9,14]. For analysis of radioactive fatty acids, 100 μl of a culture grown overnight in LB medium was transferred into 5 ml of RB medium supplemented with 0.1% *cis*-9, 10-methylenehexadecanoic acid (a cyclopropane fatty acid) plus 0.01% L-arabinose. After incubation of these cultures for 1 h, 5 μCi of sodium [1-^14^C] acetate was added and the culture allowed continuing growth for 4 h. The phospholipids were then extracted as described above. The phospholipid acyl chains were converted to their methyl esters, which were separated by argentation thin-layer chromatography, and analyzed with autoradiography [12]

### Expression of plasmid-encoded proteins

To assay expression of the products of *C. acetobutylicium fabF1 *and *fabZ*, pHW28 and pHW39 were introduced into *E. coli *strain BL21 (DE3), which encodes T7 RNA polymerase under the control of the IPTG-inducible *lacUV5 *promoter. The products of the cloned gene were selectively labeled with [^35^S]methionine as described [32]. The proteins were separated on a sodium dodecyl sulfate-12% polyacrylamide gel (pH 8.8). The destained gels were dried, and the labeled proteins were visualized by autoradiography [32]. The molecular mass markers (Bio-Rad, Richmond, Calif) were rabbit phosphorylase, bovine serum albumin, rabbit actin, bovine carbonic anhydrase, trypsin inhibitor and hen egg white lysozyme.

### Purification of FabF1 and FabZ

Plasmid pHW76 and pHW74m were introduced into strain BL21 (DE3), respectively, and the proteins were overexpressed and purified as described previously[20]. The enzymes were homogeneous as judged by sodium dodecyl sulfate-polyacrylamide gel electrophoresis. The *E. coli *FabD, FabH, FabG, FabA, FabZ, FabB, FabI and *Vibrio harveyi *AasS proteins were purified by their hexahisitidine tags described previously [18,20].

### Assay of FabF1 and FabZ activity in *vitro*

Fatty acid synthesis was reconstituted in *vitro *to assay FabF1 and FabZ activity using the purified enzymes that catalyze the fatty acid biosynthesis essentially. The assay utilized the AasS acyl-ACP synthetase from *Vibrio harveyi *[18] to generate 3-hydroxydecanoyl-ACP. The reaction mixtures to synthesize 3-hydroxydecanoyl-ACP contained 20 μM ACP, 10 mM ATP, 10 mM MgSO_4_, 5 mM DTT, 0.1 M sodium phosphate buffer (pH 7.0), 100 μM 3-hydroxydecanoic acid (Sigma) and AasS (0.2 μg) in a final volume of 50 μl. The mixtures were incubated at 37°C for 1 h. To assay *C. acetobutylicium *FabF1, the following incubation 1 μg each of *E. coli *FabD, FabG and FabA, 100 μM NADPH, 100 μM NADH, 100 μM malonyl-CoA, and 1 μg of either *E. coli *FabB or *C. acetobutylicium *FabF1 was added. To assay *C. acetobutylicium *FabZ, the following incubation contained 1 μg each of *E. coli *FabD, FabG and FabB, 100 μM NADPH, 100 μM NADH, 100 μM malonyl-CoA, and 1 μg of *E. coli *FabA or *C. acetobutylicium *FabZ was added. The resulting mixture was incubated for an additional 1 h and the reaction products were analyzed by conformationally sensitive gel electrophoresis on 20% polyacrylamide gels containing 2.5 M urea [20,24]. The gel was stained with Coomassie Brilliant Blue R250.

## Authors' contributions

LZ cloned *Clostridium acetobutylicium fabF*s genes, constructed several *fabF *expression vectors and did complementation experiments with *fabF*s expression vectors. JC cloned *Clostridium acetobutylicium fabZ *gene and made *E. coli fabZ *mutant. BL changed codons that correspond to rare *E. coli *tRNA species in *C. acetobutylicium fabZ *to codons favored in *E. coli *by site-directed mutagenesis. SF carried out biochemical studies on FabF and FabZ of *C. acetobutylicium *in *vitro*. JL performed expression experiments and purified FabF and FabZ proteins. SW helped to design the PCR primers. JEC participated in the design of the study and helped to draft the manuscript. HW conceived of the study, and participated in its design and coordination and helped to draft the manuscript. All authors read and approved the final manuscript.

## Supplementary Material

Additional file 1 **Bacterial strains, plasmids and oligonucleotides used in this work**. The data provided bacteria strains, plasmids and oligonucleotides used in this work. Click here for file
