# Supplementary material for: Functions of the Clostridium acetobutylicium FabF and FabZ proteins in unsaturated fatty acid biosynthesis
Source: BMC Microbiol. 2009 Jun 4;9:119. doi: 10.1186/1471-2180-9-119 (PMC2700279; doi:10.1186/1471-2180-9-119)
Supplement: Additional file 1 — Bacterial strains, plasmids and oligonucleotides used in this work. The data provided bacteria strains, plasmids and oligonucleotides used in this work. [file 1471-2180-9-119-S1.pdf]

Strains or plasmids	Relevant characteristics	Source or reference
Strains		
DH5α	F'φ80Δ*lacZ*ΔM15/Δ(*lacZYA-argF*)*U*169*recAendA*1 *hasR*17	Laboratory collection
CY244	*fabB15*(Ts)*fabF*	[12]
JWC275	*fabB15*(Ts) *fabF::kan*	[33]
CY242	*fabB15*(Ts)	[12]
K1060	*fabB*5	[34]
CY57	*fabA*2(Ts)	Laboratory collection
MH121	*fabA*::*lacZ*	Laboratory collection
BL21(DE3)	*E. coli* B F^- *ompT* $r_B^- m_B^-$ (λDE3)	Laboratory collection
DY330	W3110 Δ*lacU*169 *gal*490 λcI 857Δ(*cro-bioA*)	Laboratory collection
MG1655	*rph*-1 *fnr*(?)	Laboratory collection
MR52	Km^r, *fabF::kan*	[35]
HW7	*E. coli fabZ* strain arrying *C. acetobutylicium fabZ*-encoding plasmid, pHW22	This work
Plasmids		
pCR2.1	Amp^r, Km^r, TA colning vector	Invitrogen
pBAD24	Amp^r, expression vector	[30]
pKD13	Template vector	[31]
pHSG576	Cm^r, cloning vector, low-copy-number plasmid	[28]
pSU20	Cm^r, cloning vector, medium-copy-number plasmid	[29]
pET28b	Km^r, expression vector	Novagen
pHW15	Amp^r, Km^r, PCR-amplified *fabZ* from *C. acetobutylicium* genomic DNA and cloned into pCR2.1 vector	This work
pHW16	Amp^r, Km^r, PCR-amplified *fabF1*from pHW33	This work

	and cloned into pCR2.1 vector	
pHW22	Amp^r, BspLU11I-HindIII fragment from pHW15 cloned into the NcoI and HindIII sites of pBAD24	This work
pHW22m	Amp^r, NcoI-HindIII fragment from pHW74m cloned into NcoI and HindIII sites of pBAD24	This work
pHW28	Km^r, BspHI-EcoRI fragment from pHW16 cloned into NcoI and EcoRI sites of pET28b.	This work
pHW76	Km^r, NdeI-EcoRI fragment from pHW28 inserted into the NdeI and EcoRI sites of pET28b	This work
pHW31	Amp^r, Km^r, PCR-amplified *fabF*2from pHW34 and inserted into pCR2.1 vector	This work
pHW32	Amp^r, Km^r, PCR-amplified *fabF*3from pHW35 and inserted into pCR2.1 vector	This work
pHW33	Cm^r, EcoRI fragment of pHW40 inserted into the same site of pHSG576.	This work
pHW34	Cm^r, HindIII-S*al*I fragment of pHW43 transferred to the same sites of pHSG576	This work
pHW35	Cm^r, EcoRI fragment of pHW42 inserted into the same site of pHSG576.	This work
pHW36	Amp^r, NcoI-HindIII fragment from pHW16 cloned into same sites of pBAD24	This work
pHW37	Amp^r, BspHI-PstI fragment from pHW31 cloned into the NcoI and PstI sites of pBAD24	This work
pHW38	Amp^r, BspHI-PstI fragment from pHW32 cloned into the NcoI and PstI sites of pBAD24	This work
pHW39	Km^r, BspLU11I-EcoRI fragment from pHW15 cloned into the NcoI and EcoRI sites of pET28b.	This work
pHW40	Amp^r, Km^r, PCR-amplified *fabF1*from *C.*	This work

	acetobutylicium genomic DNA and cloned into pCR2.1 vector	
pHW41	Amp^r, Km^r, PCR-amplified *fabF2*from *C. acetobutylicium* genomic DNA and cloned into pCR2.1 vector	This work
pHW42	Amp^r, Km^r, PCR-amplified *fabF*3from *C. acetobutylicium* genomic DNA and cloned into pCR2.1 vector	This work
pHW43	Cm^r, *Hind*III-*Xho*I fragment of pHW41 inserted between the same sites of pSU20	This work
pHW74	Km^r, NdeI-EcoRI fragment from pHW39 inserted into the NdeI and EcoRI sites of pET28b.	This work
pHW74m	Codon optimized *fabZ* of pHW74	This work

Oligonucleotides

Name	Sequence (5'-3')	Cut Site
*fabF*1		
primerF1	TTGTAAAGGAAGGTGCAGTAAATGAATAGG	
primerF2	CCATGTTATACAACACTTCCTTCTCAATT	
primer12	AGGTGCAGTtcATGAATAGG	BspHI
F1ndeI1	CTTTAAGAAGGAGATATACATCATATGAATAGG AGAGTTGTTATAAC	NdeI
F1ndeI2	GTTATAACAACTCTCCTATTCATATGATGTATAT CTCCTTCTTAAAG	NdeI
*fabF*2		
primerF3	ATTAAGTAGGAGTATGATAAAATGGAAAGC	
primerF4	GTAAGTTTTCCGGCAGATGGCCATTTAAA	
primer34	AGTATGATACCATGGAAAGC	NcoI

*fabF*3		
primerF5	ATCGTATGAGGAGGTGTTAGAATGAGGAGA	
primerF6	TTGGATTTAATATTCCATAGCTGGATTCCA	
primer56	GAGGTGTTATCATGAGGAGA	BspHI
fabZ		
Zprimer1	GTAAATTGAAAGCACATGTTTTTATTG	
Zprimer2	CTAGGATTACTGAAGCGAGTG	
Z ndeI1	CTTTAAGAAGGAGATATACATATGTTTTTATTGAGTTTAAGTATAG	NdeI
Z ndeI2	CTATACTTAAACTCAATAAAAACATATGTATATCTCCTTCTTAAAG	NdeI
E.coli fabZ deletion primers		
HZ1	ccgtgttattattgtcgtttcttatattttgacaggaagagtatcTGTGTAGGCTGGAGCTGCTTCGA	
HZ2	cacaatggcggttggatgcacaaaggcggatttatcaatcacgtaCATATGAATATCCTCCTTAGTT	
P1	AAGCGTCTGAATTCGCTTGA	
P2	CGACATGGGATCCACCGATA	
Primers of ca *fabZ* mutagenesis		
11	GTTTAAGTATTGAACAAATTATGGAAATTATTCCTCATCGCTATCCAATG	
12	CATTGGATAGCGATGAGGAATAATTTCCATAATTTGTTCAATACTTAAAC	
21	GGTAGACcGtGTTGAAGAAATtGAACCAGGGAAAcGtGCAGTGGGcTATAAAAATG	
22	CATTTTTATAgCCCACTGCaCgTTTCCCTGGTTCaATTTCTTCAACaCgGTCTACC	
31	GAACAGATcTTTCAAGGTCATTACCCAGGTAAACCAATtATGCCAGGtGTTCTTATG	

32	CATAAGAACaCCTGGCATaATTGGTTTACCTGGG TAATGACCTTGAAAgATCTGTTC
41	GATAAGTATAAGGGtAAGAAGCCTATtCTTGGtGC AGTAAAGAATGC
42	GCATTCTTTACTGCaCCAAGaATAGGCTTCTTaCC CTTATACTTATC
51	GAATGCTAAGTTTAGtAGcATGGTAGTTCCAGGT GATGTACTgAAGTTAGAAATTG
52	CAATTTCTAACTTcAGTACATCACCTGGAACTAC CATgCTaCTAAACTTAGCATTC
61	GAAATTGAAATtGTTAAAGTTAAAGGtCCAGCTG GcATtGGtAAAGGtATtGCAACGG
62	CCGTTGCaATaCCTTTaCCaATgCCAGCTGGaCCTT TAACTTTAACaATTTCAATTTC
71	5’GGTTAATGGtGAAAAGGCAGTAGAAGCTGAAA TTACATTTATGATtGTATAG
72	CCGTTGCaATaCCTTTaCCaATgCCAGCTGGaCCTT TAACTTTAACaATTTCAATTTC
